# Supplementary material for: Change in Shoulder Function in the Early Recovery Phase after Breast Cancer Surgery: A Prospective Observational Study
Source: J Clin Med. 2021 Jul 31;10(15):3416. doi: 10.3390/jcm10153416 (PMC8347494; doi:10.3390/jcm10153416)
Supplement: Supplementary file 1 [file jcm-10-03416-s001.zip › jcm-1295328-supplementary.pptx]

## Slide 1
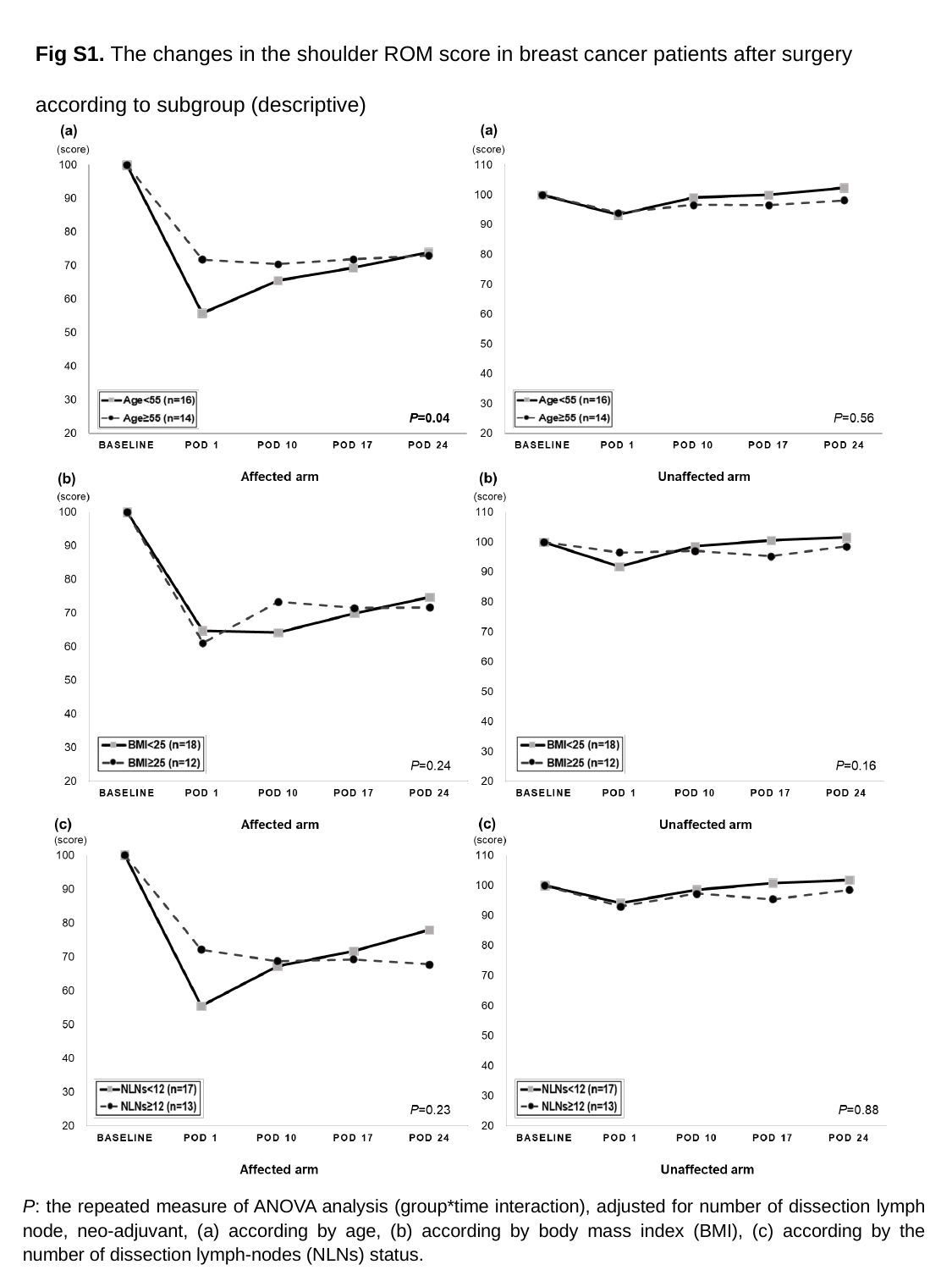

Fig S1. The changes in the shoulder ROM score in breast cancer patients after surgery according to subgroup (descriptive)
P: the repeated measure of ANOVA analysis (group*time interaction), adjusted for number of dissection lymph node, neo-adjuvant, (a) according by age, (b) according by body mass index (BMI), (c) according by the number of dissection lymph-nodes (NLNs) status.

## Slide 2
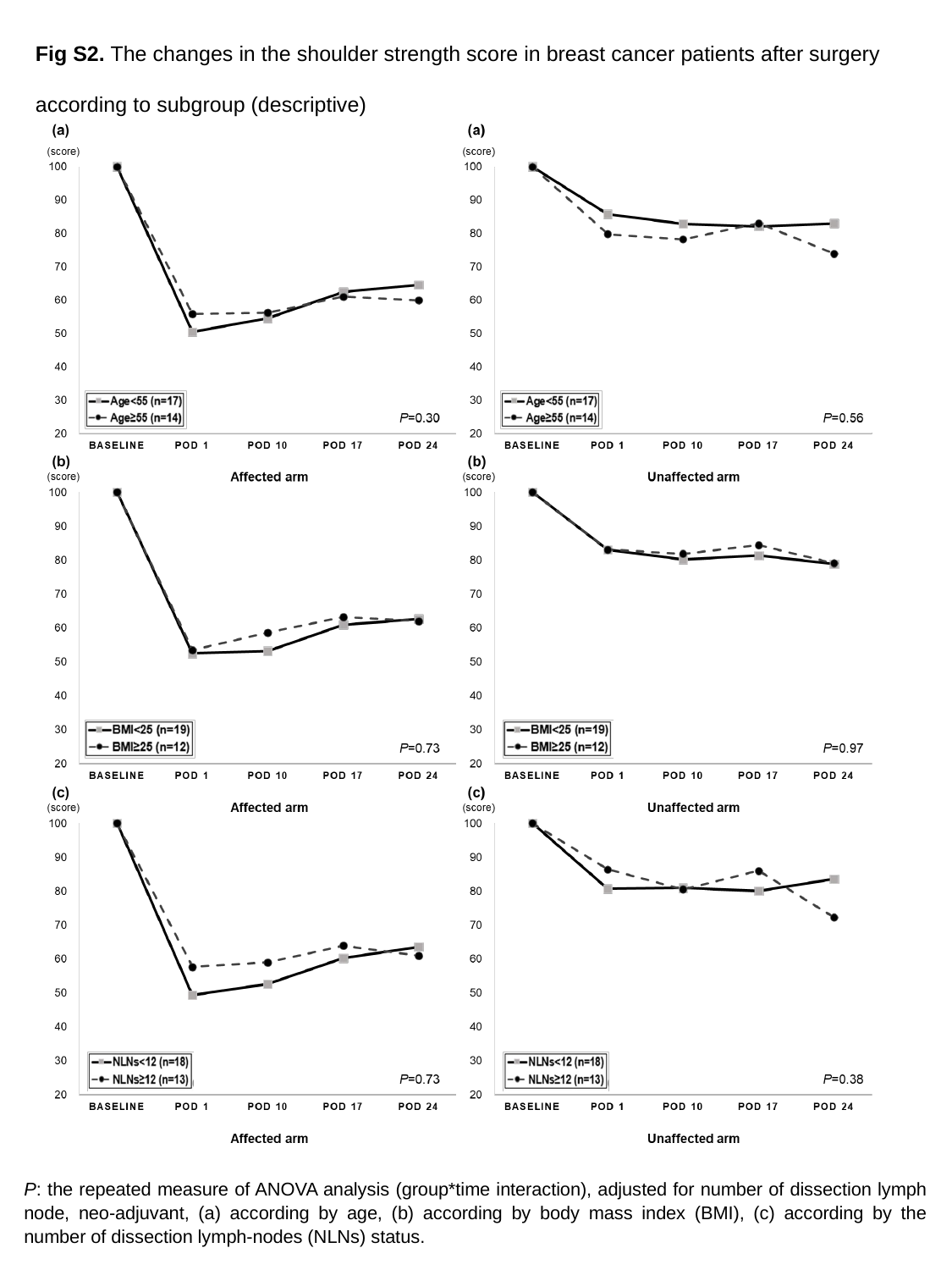

Fig S2. The changes in the shoulder strength score in breast cancer patients after surgery according to subgroup (descriptive)
P: the repeated measure of ANOVA analysis (group*time interaction), adjusted for number of dissection lymph node, neo-adjuvant, (a) according by age, (b) according by body mass index (BMI), (c) according by the number of dissection lymph-nodes (NLNs) status.
